# Clozapine initiation in the Belfast Health and Social Care Trust (BHSCT)

**DOI:** 10.1192/bjo.2021.482

**Published:** 2021-06-18

**Authors:** Rebecca Cairns, Stephen Guy

**Affiliations:** Belfast Health and Social Care Trust

## Abstract

**Aims:**

The aim of this project is to improve the quality of documentation and recording of the assessment and monitoring of patients commencing clozapine in BHSCT.

**Background:**

Clozapine is an effective treatment for patients with schizophrenia who have not responded to at least two other antipsychotics. Due to clozapine's significant side effect profile patients must be carefully assessed prior to treatment initiation with close monitoring of their physical observations and reported side effects during initiation.

The BHSCT Clozapine Pathway currently uses a Clozapine Assessment Integrated Care Pathway (ICP) common to inpatient and outpatient clozapine titrations and a Clozapine Titration ICP which varies slightly between inpatient and outpatient titrations.

**Method:**

The Clozapine ICPs of patients commenced on clozapine in BHSCT in a 9 month period commencing January 2019 were reviewed. Handwritten clinical records were used to collect data on rates of completion of all aspects of the pathway.

These results were used to identify areas of the pathway that were being poorly completed and the “Method for Improvement Model” used to trial changes to the pathway using Plan-Do-Study-Act (PDSA) cycles.

**Result:**

20 patients in BHSCT were commenced on clozapine in the 9 month period. 1 Clozapine Initiation Pathway could not be located; therefore data were collected on 19 patients. 2 patients were initiated in the community and 17 patients initiated as inpatients.

The results showed that sections of the Clozapine Assessment ICP were poorly completed; for example only 27% of the “Patient Baseline Preparation Checklist” were complete, with 60% partially complete and 13% completely blank.

In the inpatient clozapine titration ICP the physical observations record was complete in only 20% of patients and the side effects monitoring record complete in only 13% of patients. Conversely the physical observations and side effects monitoring records were complete in 100% (n = 2) of patients.

**Conclusion:**

BHSCT Clozapine Pathways were being poorly completed, with outpatient pathways being completed better than inpatient pathways. Analysis of the data shows that repetition of information in various parts of the pathway leads to gaps in documentation.

Parts of the pathway that were poorly completed have been redesigned and the impact of these changes assessed using the PDSA cycle method. It is hoped that this along with education of staff will lead to an improvement in the assessment and monitoring of patients being commenced on clozapine.

